# Supplementary material for: Transcriptome Analysis Reveals Vimentin-Induced Disruption of Cell–Cell Associations Augments Breast Cancer Cell Migration
Source: Cells. 2022 Dec 13;11(24):4035. doi: 10.3390/cells11244035 (PMC9776984; doi:10.3390/cells11244035)
Supplement: Supplementary file 1 [file cells-11-04035-s001.zip › DEGs and enrichment analysis.zip!/DEGs and enrichment analysis/WT_VIMvsVector GO enrichment/UP/WT_VIMvsVector_up.GOmf_DAG.pdf]

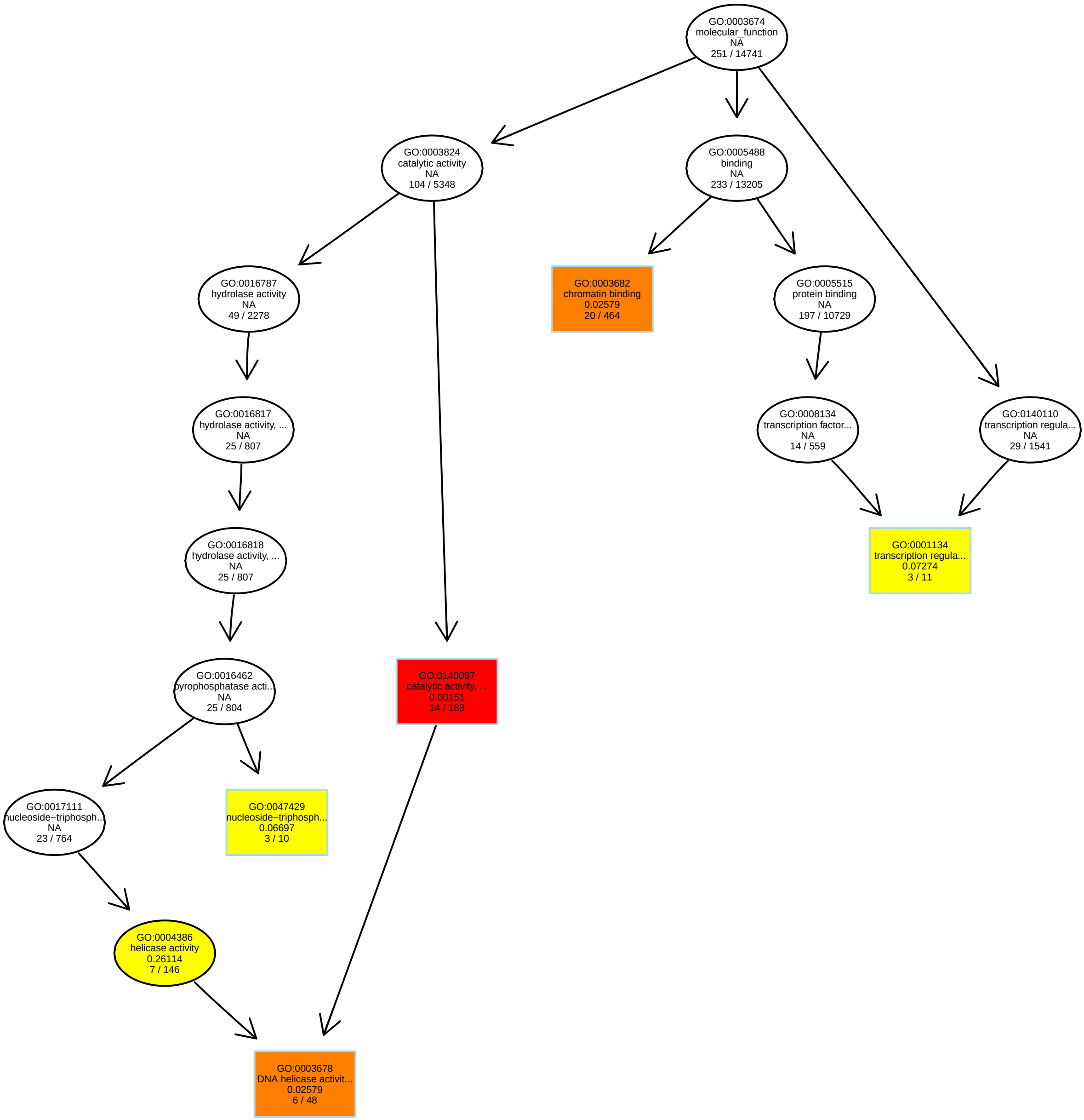
GO:0003674
molecular_function
NA
251 / 14741
GO:0003824
catalytic activity
NA
104 / 5348
GO:0005488
binding
NA
233 / 13205
GO:0016787
hydrolase activity
NA
49 / 2278
GO:0003682
chromatin binding
0.02579
20 / 464
GO:0005515
protein binding
NA
197 / 10729
GO:0016817
hydrolase activity, ...
NA
25 / 807
GO:0008134
transcription factor...
NA
14 / 559
GO:0140110
transcription regula...
NA
29 / 1541
GO:0016818
hydrolase activity, ...
NA
25 / 807
GO:0001134
transcription regula...
0.07274
3 / 11
GO:0016462
pyrophosphatase acti...
NA
25 / 804
GO:0140097
catalytic activity, ...
0.00151
14 / 183
GO:0017111
nucleoside−triphosph...
NA
23 / 764
GO:0047429
nucleoside−triphosph...
0.06697
3 / 10
GO:0004386
helicase activity
0.26114
7 / 146
GO:0003678
DNA helicase activit...
0.02579
6 / 48
